# Focal Dome Osteotomy for the Treatment of Diaphyseal Malunion of the Lower Extremity

**DOI:** 10.3390/medicina58020308

**Published:** 2022-02-17

**Authors:** Rafael Neiman

**Affiliations:** Orthopedic Trauma Surgeons of Northern California, Carmichael, CA 95608, USA; rafaelneiman@gmail.com

**Keywords:** malunion, osteotomy, focal dome, diaphysis, deformity correction

## Abstract

The treatment of malunion of the lower extremity diaphysis is challenging. Diaphyseal osteotomies require extra care to promote bone healing. This may be enhanced through osteotomies, which do not produce bone gaps and allow for compression. The focal dome osteotomy allows for rotation around an axis to correct angular deformity. The production of a successful arcuate or focal dome osteotomy requires a suitable soft tissue host. The deformity analysis is not complex but essential to assess the feasibility of correction and is required for perfect execution of the osteotomy. This tutorial explains the technique for focal dome osteotomy to correct angular deformities of the lower extremities, specifically in the diaphysis. Surgical correction for malunion, infected malunion, and infected mal-nonunion case examples are discussed. With meticulous planning and surgical technique, the focal dome osteotomy is a viable option for correcting diaphyseal malunions with compression techniques that allow a stable construct for early weight-bearing.

## 1. Introduction

Malunions have always challenged orthopedic surgeons, with clinicians employing varying techniques, exposures, and fixation methods. The correction of a malunion ranges from simple to complex and from acute to progressive, with differing levels of success. When sound principles are applied, generally good, predictable results can be expected [1].

The correction of lower extremity malunion requires multiple considerations in order for a successful outcome to occur. The patient must have adequate soft tissue to handle the correction, the malunion must be understood completely so that correction can be achieved with the simplest yet most effective means, and stability must be imparted in a logical fashion with internal or external fixation in order to maintain the correction. Corrections can be achieved through acute or progressive corrections. Acute corrections have more constraints regarding soft tissue risk. Slow progressive corrections require unique challenges with regards to the means of fixation, along with patient participation and compliance.

**Figure 1:**
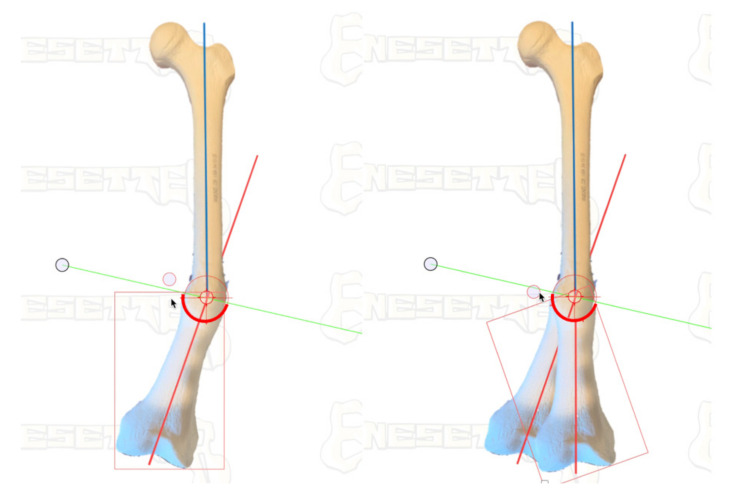
Focal Dome Osteotomy Concept. The Center of Rotation of Angulation (CORA) is the intersection of proximal and distal anatomical axes through a deformity. Focal Dome Osteotomy is an arcuate osteotomy based on a semicircular cut centered at and rotated about the CORA. The distal femur in this example rotates around CORA, fully correcting the deformity (blue and red axis lines become collinear).

This tutorial describes the author’s technique for acute correction of lower extremity diaphyseal malunions using an arcuate or focal dome osteotomy. A focal dome osteotomy is a semicircular osteotomy based upon an arc drawn from the radius about a central point (Figure 1). This central point is placed at the center of rotation of angulation (CORA) [2]. CORA is determined by the intersection of the lines drawn through the central anatomic axis of the proximal and distal diaphyseal segments of the bone in question. The focal dome osteotomy requires understanding and calculating the plane of deformity, and its execution can be unforgiving, especially in cortical bone. With proper technique, this osteotomy results in a compressible correction with little to no bone void, allowing for early or immediate weight-bearing. The author will also describe a technique for the application of a true dome osteotomy, which can be used to correct multiplanar deformities, including rotational deformities, though, currently, its application is limited to select indications due to the requirements of wider exposure and greater soft tissue stripping.

### General Concepts of Lower Extremity Correction and Indications for Focal Dome Osteotomy

It is beyond scope of this paper to recommend specific angular parameters as a threshold of when to perform a corrective osteotomy. Once the decision has been made to execute a lower extremity deformity correction, the varying types of corrections must be considered. Malunion correction is generally divided into subcategories of acute and progressive corrections, and within each of these corrections, the surgeon will decide upon those that use an opening wedge, closing wedge, sliding/oblique osteotomies, clamshell osteotomies, or, as the focus of this tutorial, focal dome osteotomy. Progressive corrections generally use a method of slow distraction using external fixation. In addition to universal application by certain surgeons for all corrections, gradual corrections are indicated in regions of poor soft tissue envelopes not capable of sustaining surgical insult, rapid correction, or internal fixation. 

Diaphyseal and metaphyseal deformities influence the decision for the type of osteotomy, where fixed surgical gaps become significantly more tolerable in metaphyseal bone than in dense cortical bone and heal more rapidly than in cortical bone [3]. Closing and opening wedge osteotomies are common in the metaphysis, as the execution is relatively simple and the technique forgiving. The gaps created in the metaphysis of a stable construct generally heal predictably [1,4]. In contrast, diaphyseal gaps are not well-tolerated. Choosing a diaphyseal location for osteotomy requires considerably more forethought. The deformity is often at a level of prior fracture. There may no longer be an intramedullary canal, which influences bone healing and fixation choice. Prior injury and/or fixation may have resulted in periosteal damage [5]. When choosing a diaphyseal osteotomy under these conditions, the fixation must be very stable and preferably compressed. The focal dome osteotomy contributes to the inherent stability of the construct due to its shape.

The morphology of a deformity is also important to consider when planning an osteotomy. A short acute bend will allow options not necessarily available when the malunion extends for a longer distance along the bone. For example, an acute focal bend can be treated with a focal dome with little residual deformity, allowing for nearly any form of internal fixation. Conversely, that same osteotomy placed along the center of a very long deformity will still have curved bone segments proximal and distal to the cut, challenging the use of an intramedullary device for stabilization. In these cases, a clamshell osteotomy may be the wisest choice [6].

When an acute correction is planned in a region where an opening wedge is not tolerable, a closing wedge shortens unacceptably, while a compressible surface is desired, a focal dome osteotomy should be considered. 

Contraindications to a focal dome osteotomy include corrections that require a greater angular correction than the arc of the osteotomy can accommodate. This limitation is learned from the manual preoperative planning phase, where the correction is performed virtually and the resulting alignment confirmed. Rotational deformities are not corrected with the focal dome osteotomy. Regions of the bone with poor healing capacity should be avoided for the focal dome osteotomy. Locations where the surgical approach cannot safely be made to correct in the proper plane for the dome osteotomy require the use of a different technique for correction.

## 2. Employing the Focal Dome Osteotomy

### 2.1. Preoperative: Physical Examination and Soft Tissue Envelope

A standard orthopedic exam for lower extremity deformity complaints will also include a gait analysis, as well as a range of motion assessment of both lower extremities. Attention to joint contracture as a cause of deformity should be noted and, if possible, addressed as part of the overall deformity correction. When moving attention specifically to the area of deformity, local and systemic factors inform the surgeon’s determination of a patient as a favorable host for this procedure [7]. Soft tissue deformity accompanies all bony deformities. The more chronic in nature the malunion is, the stiffer the soft tissues become. Periosteal, fascial, and tendon structures become more fibrous, muscles atrophy, and correction of an angular deformity results in the elongation of soft tissue on one side of the deformity with shortening on the other side of the deformity. It is essential to determine whether this type of correction can even be considered with or without the aid of additional soft tissue releases. The tibia is particularly unforgiving on the medial side, and with the propensity for the limb to drift into varus, this is unfortunately the more common location for the concave side of a tibial misalignment. The ‘structure at risk’ concept as taught in distraction osteogenesis techniques is worth considering in acute corrections as well [8]. Neurovascular and other soft tissue structures can be compromised not just with surgical exposure but also with the correction. In addition to typical comorbidities such as vascular disease and diabetes, a patient’s other risks should be acknowledged in order to stratify the patient as a surgical candidate. Nutritional and metabolic assessments and smoking status should be considered. Medications such as blood thinners, steroids, or immune suppression drugs require the surgeon to pause to consider their potential interactions, as does illicit drug use. Careful examination of the patient must include an assessment of prior incisions, prior flaps, skin grafts, and function of the limb proximal and distal to the deformity. Realizing that the approach required for this osteotomy requires a wide surgical exposure, significantly more so than a percutaneous transverse osteotomy, the surgeon must assume there will be influence from the patient’s comorbidities upon the procedure. Hence, the surgeon should preoperatively identify the potential complications and which options remain for salvage of the correction should the need arise after osteotomy. If the ‘worst case scenario’ for a wound complication is expected, a flap should be performed at the time of or immediately after surgery. For example, a varus tibia malunion with adherent skin over a previously granulated wound at the location of the osteotomy should be addressed in this fashion. Densely adherent wounds directly in the path of the exposure or correction should not be subject to this open approach without soft tissue contingency plans. The surgeon must counsel the patient of this prior to surgery, especially that these complications could occur remote to the time of osteotomy. The surgeon should either be facile with the required soft tissue technique or have immediately available someone who can provide this care. Without this approach, acute correction with a focal dome should not be attempted.

### 2.2. Preoperative: Deformity Analysis

Once a patient’s soft tissue is deemed appropriate for an acute focal dome osteotomy, the deformity analysis ensues.

An ideal patient workup includes standing full-length anteroposterior and lateral projection X-rays. These allow the assessment of anatomic and mechanical axes. Leg length should be corrected with blocks to level the pelvis and knees oriented patella forward. When the patient with a deformity also has joint contracture, additional radiographs are obtained that are orthogonal to each bone (i.e., true AP of each femur and tibia). While the initial deformity analysis is done using mechanical axis deviation, once the deformity is identified as diaphyseal, the CORA method, as described by Paley, is used to characterize the deformity within the anatomical axis [2]. A physical examination is conducted simultaneously to corroborate the deformity, leg length inequality, and rotational deformity. Again, it is important to note that the focal dome osteotomy does not address rotational deformity. If the patient has a rotational deformity in addition to their angular deformity, a true dome osteotomy can accommodate both angular and rotational deformities, though, with the current instrumentation, this may be more practically handled with other osteotomies such as a wedge osteotomy, a mathematically derived single-cut osteotomy, or a clamshell osteotomy [6].

Understanding the deformity in the coronal and sagittal planes allows the surgeon to determine the true plane of an angular deformity. This should not be considered a multiplane deformity solely because it is present in both coronal and sagittal planes; there will be a single plane that is between the coronal and sagittal planes that will encompass the entire deformity, and orthogonal to this true deformity plane, there will be no deformity. This is considered the ‘no deformity view’.

**Figure 2:**
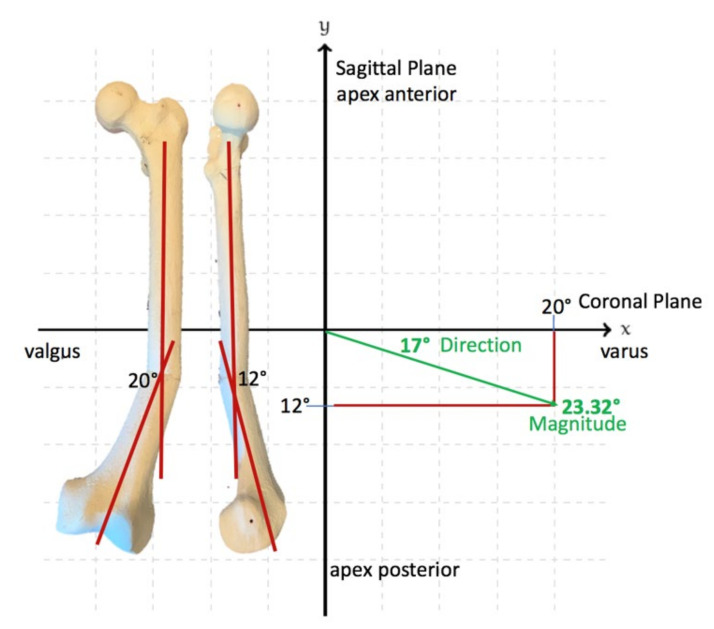
Determining the plane of the deformity. The plane of deformity can be calculated by plotting the measured deformity in the coronal and sagittal planes on the x and y axes, where the magnitude of the true deformity is the length of the resultant vector attained from the plot of the coronal and sagittal vectors. The location of the true deformity plane is the direction of this resultant vector with respect to the coronal or sagittal plane. This calculation can be done with a ruler and compass or, more accurately, using basic geometric principles. In the example shown, the maximum deformity is 23.32 degrees, and this deformity plane is located between the coronal and sagittal planes, 17 degrees from the coronal plane rotating posteriorly towards the sagittal plane.

The calculation of the true deformity and no deformity planes can easily be achieved. Once the coronal and sagittal plane deformity angles are determined, each of these values in the coronal and sagittal planes are plotted on an XY graph, as seen in Figure 2. The hypotenuse of the triangle that is created with the coronal and sagittal magnitudes is the maximum deformity in degrees. The position of this maximum deformity is also obtained from this same graphical representation. The angle that is made between either the coronal or sagittal plane is the actual plane of deformity that exists between those planes. Perpendicular to this maximum deformity plane is where the no deformity plane will be found.

Once this true plane of deformity is determined, it is correlated with a physical examination where the true deformity can be confirmed. The maximum plane of the deformity must be surgically accessible. For example, if the patient has a pure coronal plane deformity, the surgical access to this deformity would be directly anterior or directly posterior in order to perform a focal dome osteotomy. If the plane is purely in the sagittal plane, access to the deformity would be directly medial or directly lateral. The surgeon knows that a direct posterior or direct lateral approach to the mid-tibia may not be physiologically possible without likely injury to the surrounding structures. When the true deformity is somewhere between the coronal and sagittal planes, the surgeon must associate this with the patient’s soft tissue envelope to determine if the trajectory of the osteotomy is possible. Once soft tissue feasibility is confirmed, the surgeon can continue to plan the osteotomy. If the ideal location of a focal dome osteotomy cannot be safely accessed, the surgeon must consider another form of correction.

The focal dome osteotomy can be performed in one of several ways. In hard diaphyseal bone, multiple parallel drill holes can be drilled along an arc constrained by a radius at the center of rotation of angulation (CORA), obtained from preoperative planning. In softer metaphyseal bone, commercially available curved saw blades will reliably perform the focal dome osteotomy (Depuy-Synthes, West Chester, PA, USA). These saw blades do not perform well in cortical bone, as they generate heat with their fine, shallow teeth. 

**Figure 3:**
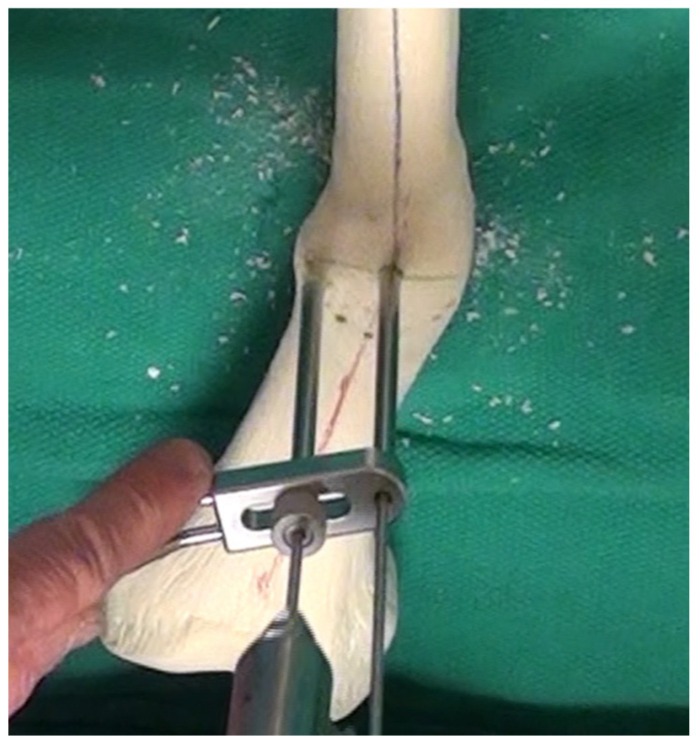
Execution of the focal dome osteotomy. A central reference drill bit is placed at the CORA. A parallel drill guide is set to the desired width, and a second drill bit is used to drill parallel holes in the bone along the arc achieved by rotating around the reference bit. Supplementary Video S1 accompanies this image.

**Figure 4:**
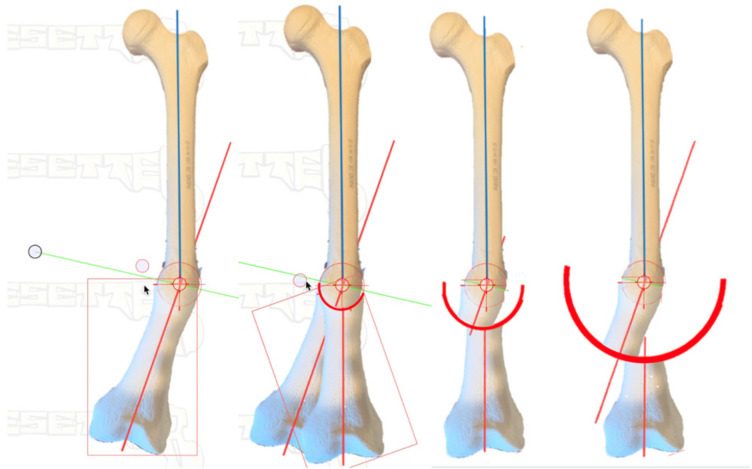
Limits of the arc of rotation for a focal dome osteotomy. When choosing increasing radii centered around the same CORA, the axial alignment will still be corrected equally in all three examples, though the remaining contact area decreases while the residual local diaphyseal deformity increases. With pure angulation deformities, the optimum radius is the smallest possible to accommodate the width of the bone at the level of the CORA.

**Figure 5:**
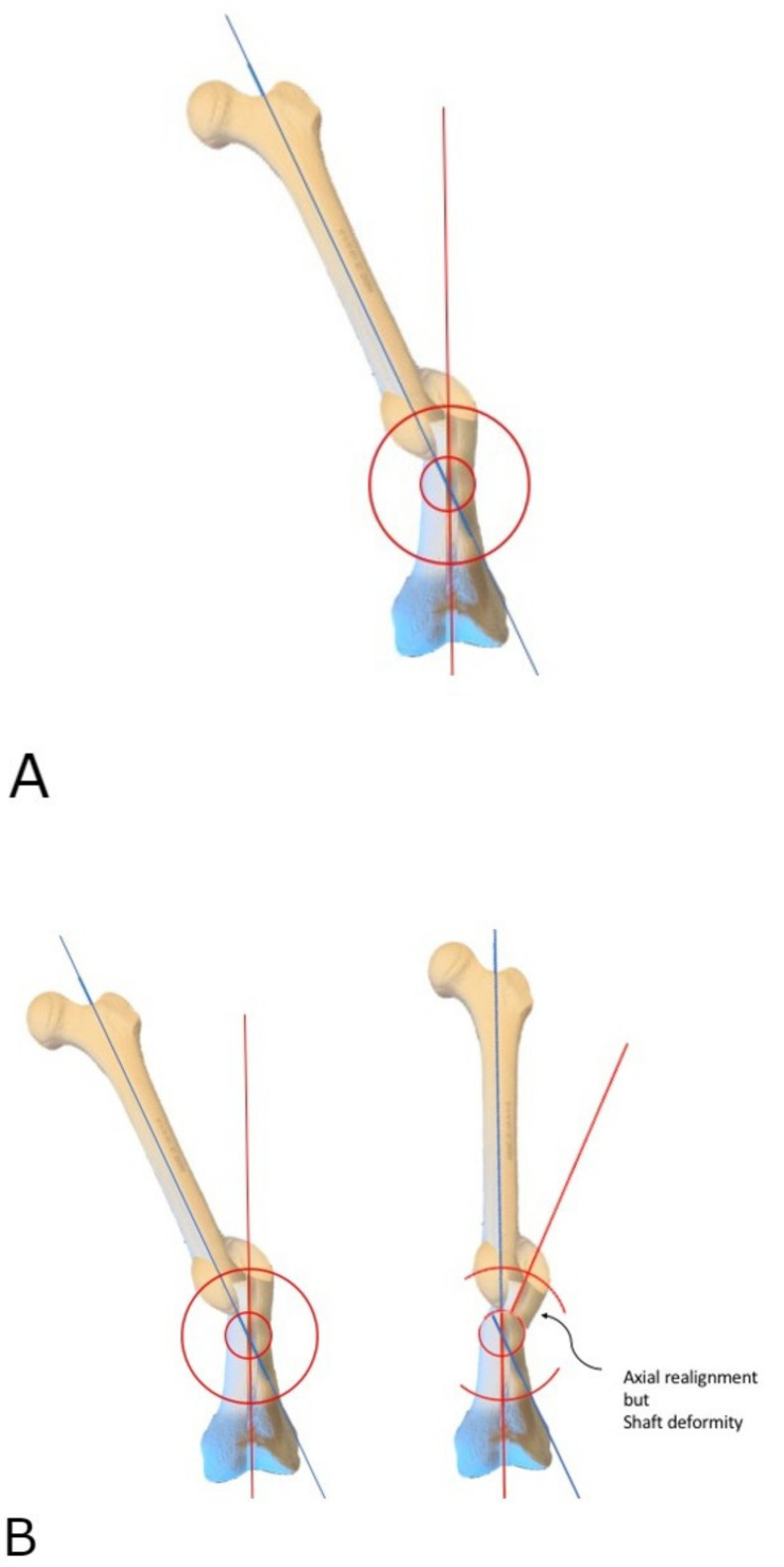

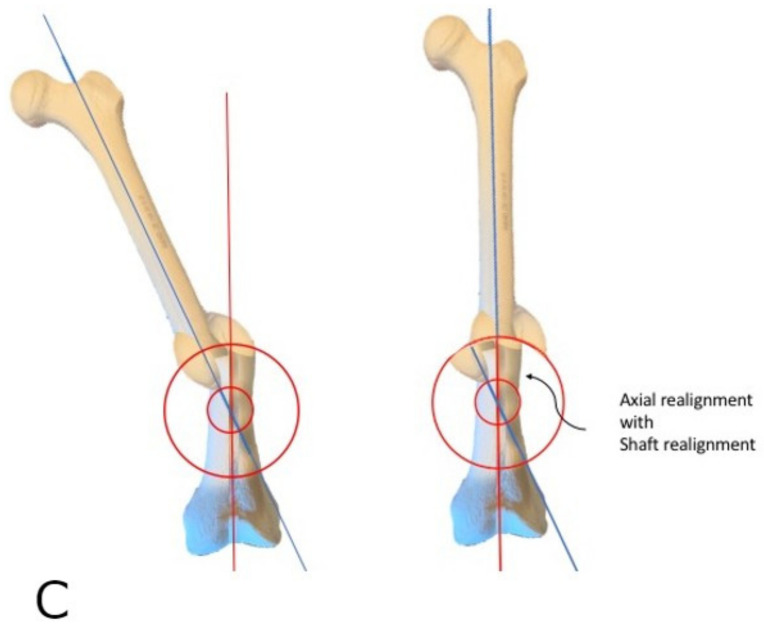
Translational deformity. When the calculation of CORA demonstrates that the intersection of the proximal and distal diaphyseal axes do not meet at the center of the deformity, this is due to translation in addition to angulation. The focal dome osteotomy can correct both the translation and angulation when the radius is centered at the CORA and not centered at the deformity. When the radius chosen for the focal dome is as small as possible, the axis is corrected, and the translation is also corrected, but the diaphysis may have some induced focal diaphyseal deformity that could prevent the use of an intramedullary implant. In contrast, when using the same CORA but enlarging the radius to reach the center of the deformity, the angular deformity and translation will correct while minimizing the local diaphyseal deformity. This is desirable for implementing intramedullary implants. (**A**) CORA demonstrates angulation, as well as translation. Two radii are shown for potential focal dome corrections. (**B**) A small radius osteotomy results in axial and translational correction, while the diaphyseal deformity that results may prevent the use of intramedullary implants. (**C**) A large radius osteotomy that coincides with the center of the deformity corrects the axis and translation while minimizing diaphyseal deformity, enabling intramedullary fixation.

Figure 3 demonstrates a safe method in cortical bone. The CORA is identified from preoperative planning using the previously determined plane and magnitude of maximum deformity. At the center of the CORA, a drill bit is placed. Based on the diameter of the bone in this location, as well as the magnitude of deformity, the radius of the curvature is chosen. With pure angulation deformities (no translation), the optimum radius is the smallest radius that also accommodates the width of the bone at that level; the closer the osteotomy is to the CORA, the more options are available for internal fixation. When the radius is elongated, this brings the osteotomy further from the CORA, which makes intramedullary fixation more of a challenge due to the local deformity, though still correcting the axial alignment. This, however, may be necessary to allow for osteotomy in healthier bone or with an improved soft tissue envelope. The benefit of focal dome osteotomy is that, provided the deformity analysis is done correctly and the CORA is established with the drill bit, the radius can be used to afford correction if there is a sufficient contact area along the cut surfaces to stabilize the correction. That is, the higher the degree of correction required, the closer the cut will need to be to the CORA (Figure 4). However, one exception is when addressing translation in addition to angulation, where larger radius focal dome osteotomy is preferred. This is discovered in the preoperative templating phase: when the CORA does not fall within the center of the deformity, translational deformity is present. This translation can be corrected with the focal dome, where CORA still defines the center, and the radius length is projected to the center of the actual deformity. Again, when the translation is also present, the osteotomy cut itself (not the CORA) is placed into the center of the deformity to minimize the residual deformity after correction (Figure 5).

### 2.3. Surgical Correction

In cortical bone, Figure 3 demonstrates a parallel drill guide used to drill multiple drill holes along the arc of the osteotomy (cannulated 6.5/7.3 screw system, Depuy-Synthes, West Chester, PA, USA). An absolutely sharp drill bit must be used and must be irrigated constantly, withdrawing the drill bit frequently to prevent overheating and damage to the cortex. This attention to detail is essential for a successful osteotomy in cortical bone. With more heat generated, more damage will occur, and the bone will require a longer healing time. This osteotomy necessitates sufficient surgical fixation and stability to resist loosening for the duration of bone healing.

Once the entire arc of the osteotomy is drilled, the space in between the drill bits can be cut using a low-energy method with a thin osteotome. This must be done extremely carefully so as to prevent escape of the osteotomy outside of the intended arc. Alternatively, a high-speed burr or small oscillating saw, also meticulously irrigated, can be used to initiate the connection between the drill holes, though completed by osteotome. Supplementary Video S1 demonstrates this technique in further detail.

Once the osteotomy is completed, the correction can be obtained. The use of the universal distractor is valuable in these cases. The intramedullary canal should also be re-established to encourage union. This must be done with as little thermal energy and soft tissue damage imparted to the cortex as possible. Manual T-handle reamers and osteotomes, as well as curettes, should be used to re-establish the canal proximal and distal to the osteotomy. If the bone is extremely dense, a saline-irrigated high-speed burr or drill may be employed to initiate the canal, as little as possible and completed with hand instruments.

Once the soft tissue has allowed for correction and the alignment is achieved using either a universal distractor or other means, compression and fixation can be applied.

With osteotomies close to the CORA, intramedullary devices should preferentially be employed. More of the implant load is shared by the bone after correction. Upon nail insertion, the distractor can be used to apply compression, or nails with compression features can be selected. Many standard nails allow for intraoperative compression, and motorized nails allow progressive or cyclic compression. If the axis of the intramedullary reamer is not sufficiently collinear with the axis of each bone segment, a gap may be produced at the osteotomy. If this occurs, three remedies can be used: (1) widen the path for the intramedullary nail and/or choose a smaller diameter nail, (2) maintain the nail position and add autologous bone graft, and (3) remove the nail and apply compression plate fixation. Factors such as malunion location, bone health, and stability required will determine which option is best. While intramedullary nails are preferred in most cases where a canal can be established, there will be occasions where extramedullary fixation, such as plates, may be preferred. When the bone is extremely sclerotic, the amount of heat generated with the osteotomy is already significant. Adding heat upon soft tissue and bone trauma in order to create a large enough pathway for nail passage should divert the surgeon to choose extramedullary fixation, provided the soft tissue in that location can handle it without additional risk of wound complications.

Once compression is applied and the fracture is absolutely stable, the osteotomy can again be assessed for any bone defects or gaps. Should there be any critical gaps felt to impair fracture healing, these are grafted with autologous bone graft.

### 2.4. True Dome Osteotomy

The true dome osteotomy is a variation of the focal dome osteotomy. This involves creating two matching concave and convex surfaces. The benefits of a true dome over a focal dome are the forgiving nature of determining the maximum plane of deformity and, especially, the ability to add rotational correction to the osteotomy [9]. The surgeon needs only to identify the location on the bone based on the CORA but not the plane and magnitude of deformity. This osteotomy provides superb stability during compression [10].

Current technology allows for applying this method in bone regions where access to the diaphysis is safe and wide enough for the placement of hemispherical concave and convex reamers [11]. 

The technique differs greatly in that after the CORA is determined, or in the case of a segmental defect where the bone end represents the CORA, a transverse osteotomy is first created, and the concave and convex reamers are then introduced on each side of the osteotomy. The reamers employed can be standard acetabular reamers to create the concave osteotomy shape, and humeral or femoral head resurfacing reamers are used for the convex surface (Aequalis, Tornier-Wright, Bloomington, MN, USA). In the case of a bulk diaphyseal graft, one side of each osteotomy is reamed ex vivo. Once each matching surface is reamed just enough to create the dome, the two surfaces are mated, and internal fixation is applied. Further reaming beyond that level only shortens the bone. Case 4 illustrates this technique. Supplementary Video S2 accompanies this manuscript to further demonstrate the concept.

### 2.5. Postoperative Treatment

In patients who have a compressed stable osteotomy, weight-bearing can be established early. When intramedullary fixation is used, surgeons can allow immediate weight-bearing.

Patients who have a significant soft tissue defect that requires local rotational or free flap coverage may have alterations in their postoperative care based on the anticipated soft tissue congestion. If the success of the flap relies upon minimizing swelling and congestion, this could significantly delay early rehabilitation; this depends entirely on the measures taken to obtain coverage.

## 3. Case Examples

### 3.1. Case 1-Multifocal Correction with Intramedullary Nail: Femur

**Figure 6:**
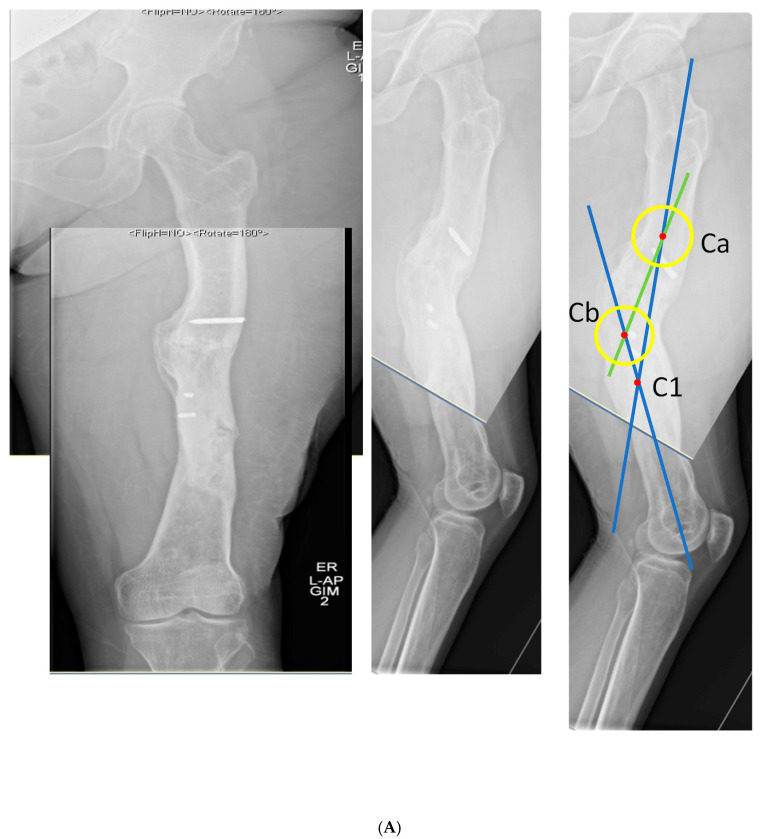

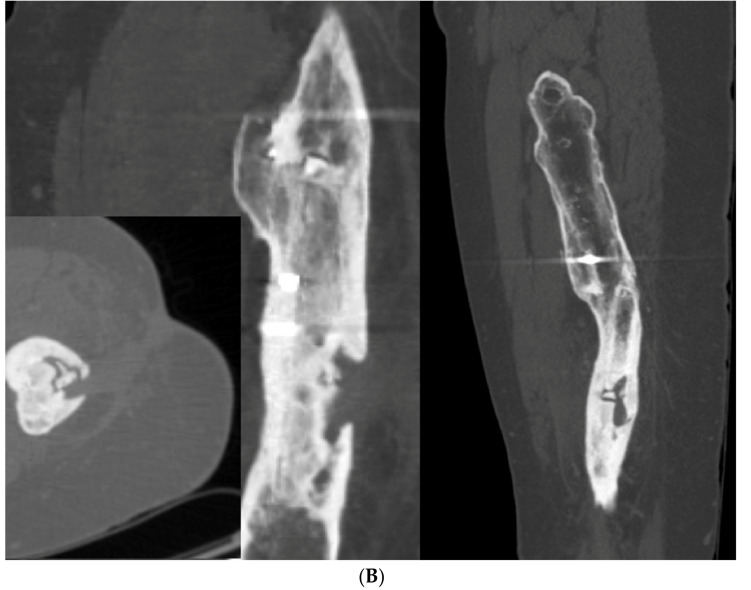

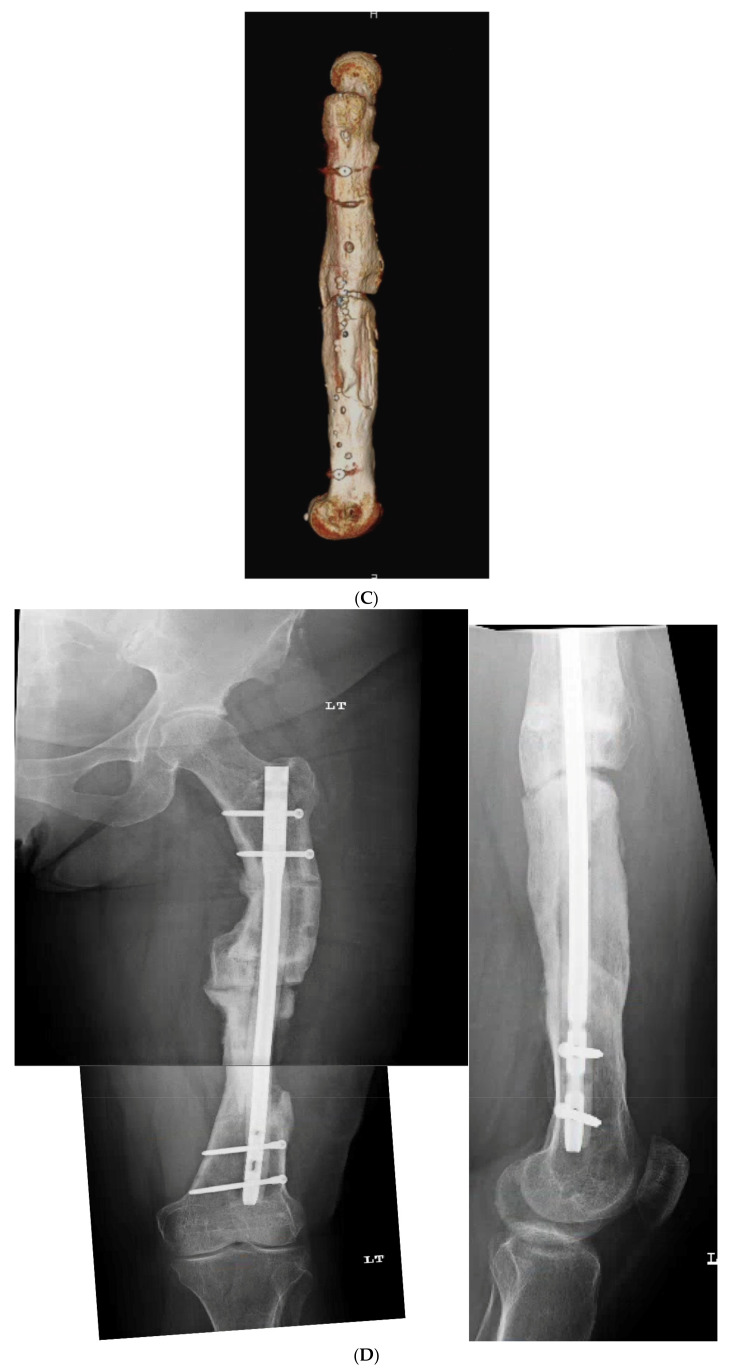

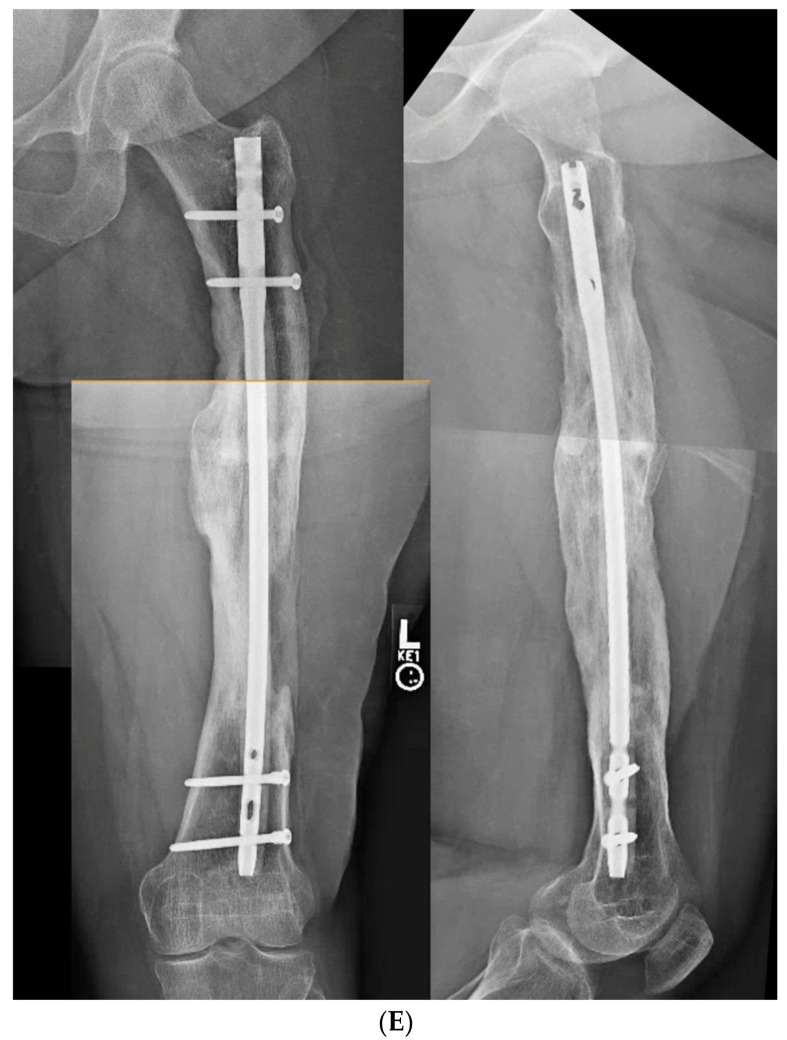
(**A**) Middle-aged female with 26 years of an infected draining sinus of the left femur with malunion and shortening. The intersection of the two blue lines represents the CORA of the proximal and distal diaphyseal segments (C1). A CORA outside of the deformity reveals translation in addition to angulation. The intersection of the green line with each blue line represents an alternative where the central deformed segment is also incorporated into the analysis, revealing two CORA center points for double-level osteotomy (Ca and Cb). (**B**) The patient was found to have retained hardware and a bone sequestrum. Sequestrectomy with antibiotic local delivery via a coated intramedullary nail with concomitant systemic antibiotics would promote infection eradication. (**C**) The patient underwent double-level focal dome osteotomy at the time of initial debridement. The patient had two sequential debridements over 10 days. Stabilization using an interlocking antibiotic coated nail was placed during the final debridement. (**D**) Five months postoperative, where the distal osteotomy was autogenous-grafted from the patient’s contralateral femur due to the persistent osteotomy gap. Chronic suppression oral antibiotics were recommended; patient voluntarily stopped antibiotics at 24 months. (**E**) Three-year final follow-up with normal serological markers and no sign of recurrence. Osteotomies healed. Soft tissue healed.

The patient is a middle-aged female with no comorbidities other than obesity who presented 26 years after an open diaphyseal femur fracture with more than 10 surgeries for attempted union and eradication of infection. The patient presented with complaints of pain at the mid-femur and knee, a chronically draining sinus of the lateral thigh, and concern for a 2-cm-short left leg. No rotational deformity is found. The limb clinically appears normal in the coronal plane. The patient has a 15-degree knee contracture. X-rays reveal a malunion of the left femur with bone sequestrum and retained hardware (Figure 6A). A metabolic workup, including the vitamin D level, is normal. The CT scan characterizes the pathological bone, allowing for precise localization of the sequestrum (Figure 6B). A deformity analysis reveals a primarily sagittal plane deformity with limb shortening. The traditional CORA analysis demonstrates translation with angulation, as demonstrated by a CORA located outside of the deformity. With the expected multiple stages, a double-level focal dome was chosen over clamshell, allowing stable weight-bearing while not requiring an extended femoral osteotomy to heal through pathological bone. The other consideration for this patient was radical en bloc resection of the diseased bone, with either bone transport or secondary reconstruction with a compressed allograft intercalary segment. This concept was reserved as the option if the current plan failed.

The patient underwent double osteotomy using focal dome osteotomy at Ca and Cb (Figure 6C), with retained hardware removal and resection of the infected sequestrum and surrounding diseased bone. A direct lateral approach to the thigh was used, and the diseased soft tissue track was excised and analyzed by pathology. A temporary nail was inserted and replaced with an antibiotic-coated interlocking nail ten days later with a planned second debridement. The nail was compressed using the internal compression instrumentation associated with the nail. A gap in the distal osteotomy persisted despite attempted removal of the nail and re-reaming with enlarging flexible reamers. This gap was accepted with intent to revisit if bone healing did not progress. The surgical soft tissue approach healed without incident. At five months postoperative, with nonunion of the distal osteotomy at the gap, the patient underwent autograft using contralateral femur reamer–irrigator–aspirator harvesting (Figure 6D). Cultures at the grafting procedure were negative.

The patient progressed to pain-free union at both osteotomies with no recurrence of her infection over three years after osteotomy (Figure 6E).

### 3.2. Case 2-Single-Plane Correction with Plate Fixation: Tibia

**Figure 7:**
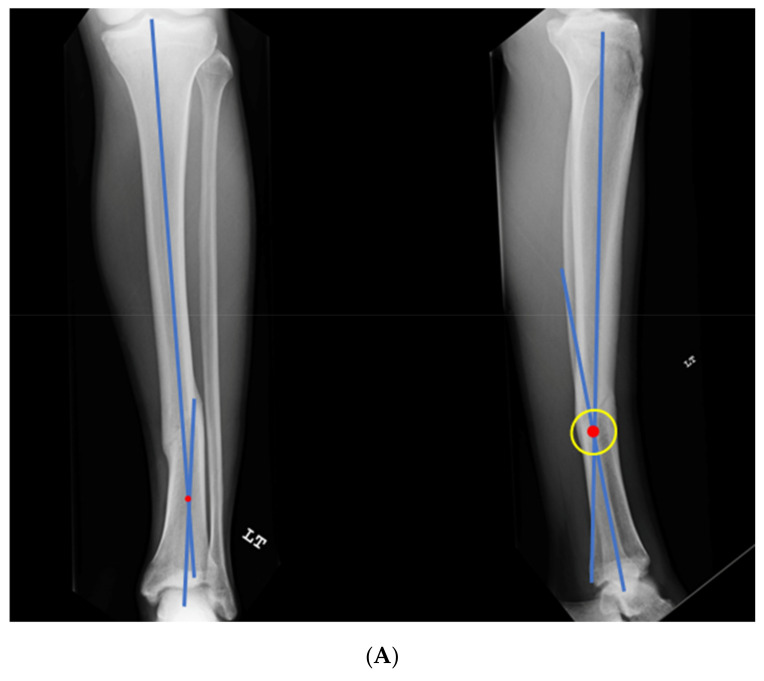

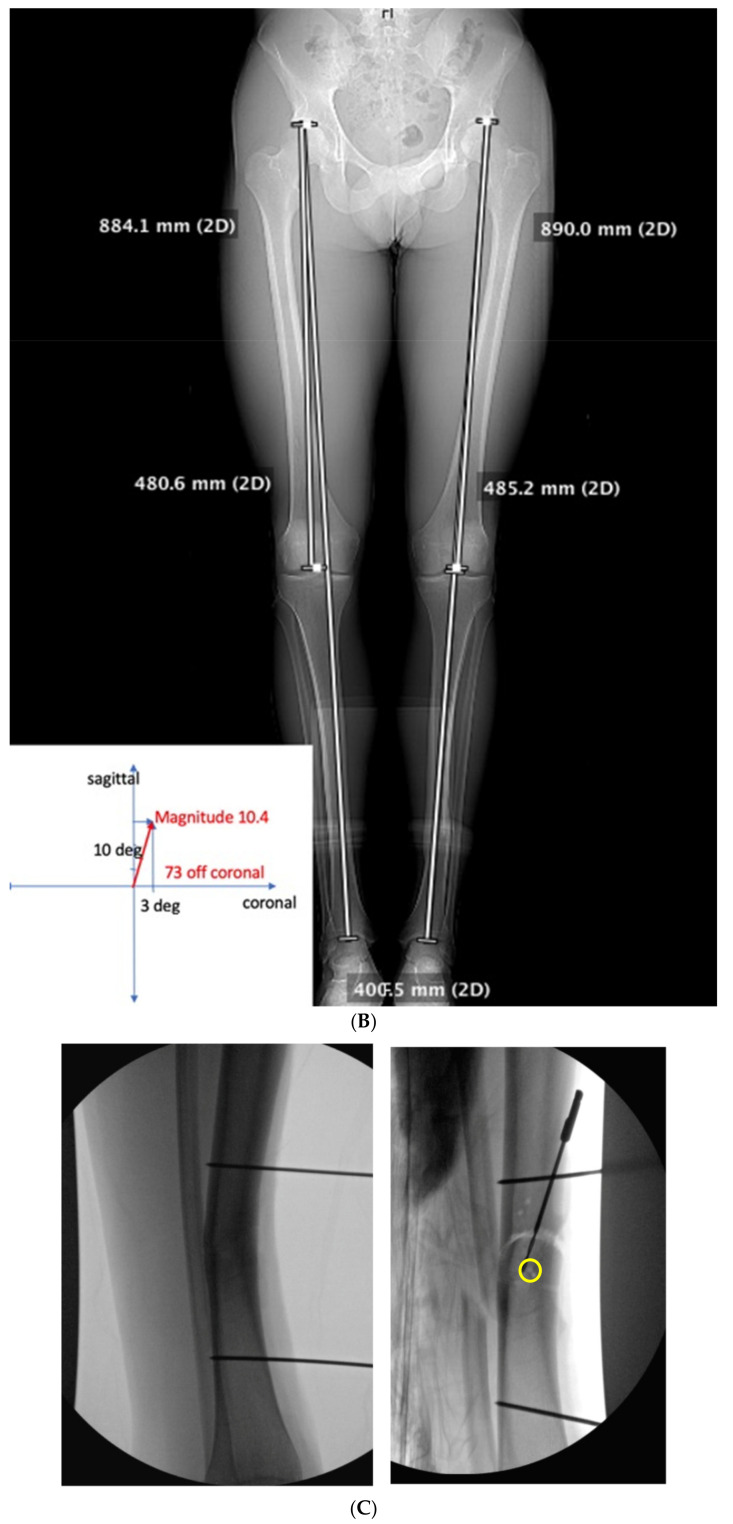

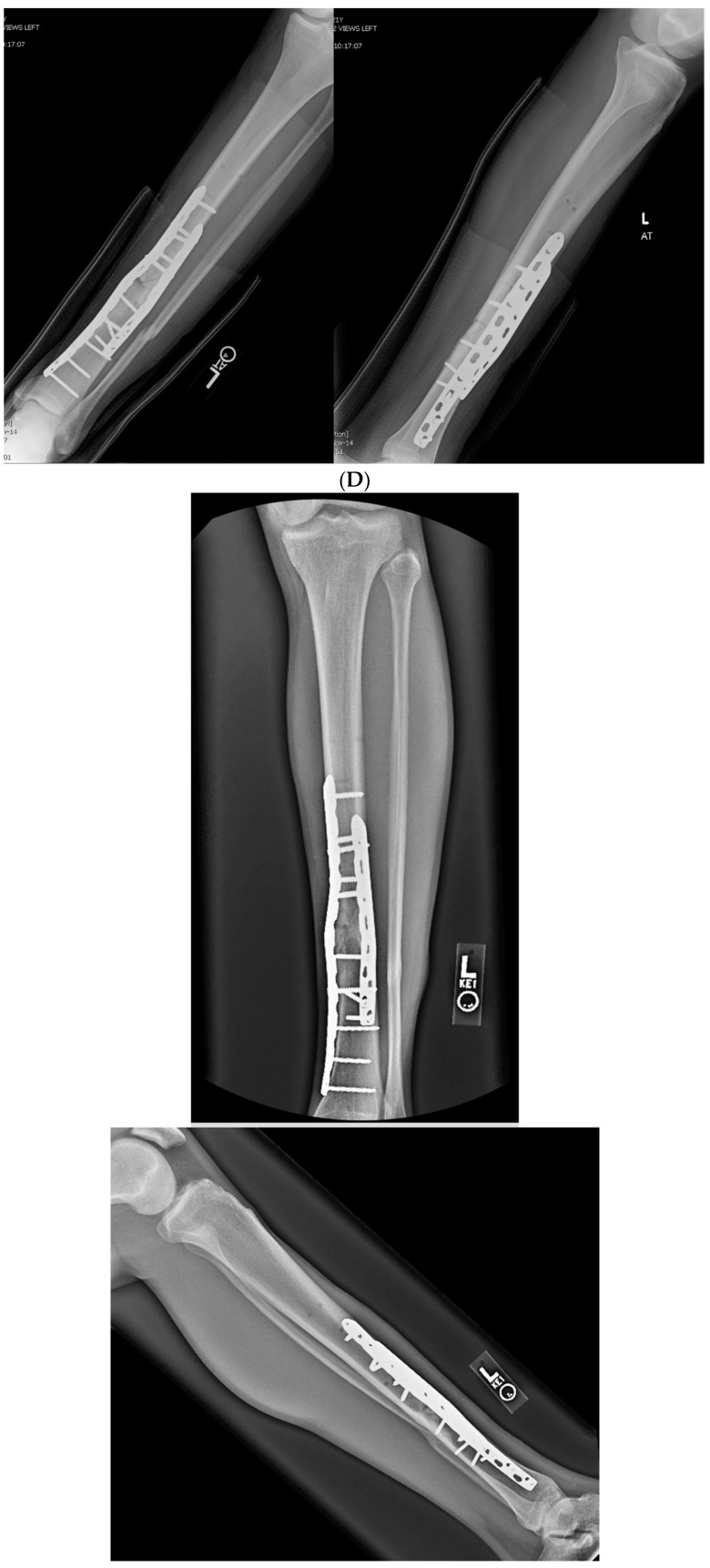

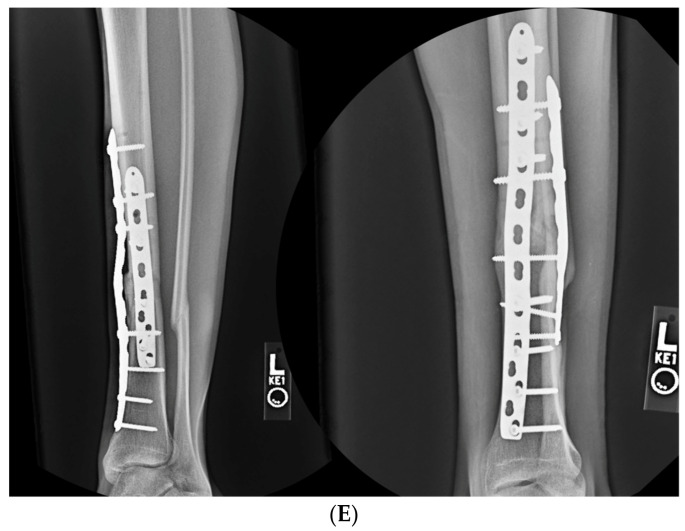
(**A**) A young adult college baseball player sustained a closed tibia fracture with intact fibula, treated closed. The patient developed a recurvatum deformity with pain at the proximal and distal tibiofibular joints. (**B**) The patient demonstrated mechanical axis lateralization due to coronal plane translation with slight varus. The plane of deformity was calculated as 73 degrees off of the coronal plane. (**C**) The patient underwent focal dome osteotomy at the CORA (yellow circle) in the plane of maximum deformity. (**D**) Dual plate compression was used. Angulation and translation were simultaneously corrected. (**E**) Final follow-up 9 months: anteroposterior, lateral, and oblique films show the osteotomy healed. Tibiofibular joint pain resolved after osteotomy. Patient returned to college-level baseball.

A young adult college baseball player presented with symptoms of lateral proximal knee pain and a feeling of knee instability 8 months after sustaining a closed fracture of the tibia, initially treated closed with casting (Figure 7A). The patient underwent a workup including full-length films, knee MRI, and local anesthetic test injection of the proximal tibiofibular joint (relieving knee pain). The CORA analysis revealed a recurvatum deformity of 10 degrees and varus deformity of 3 degrees, with translation in the coronal plane. Full-length alignment film of the pelvis and lower extremity confirmed coronal translation with mild varus angulation (Figure 7B). The true plane of deformity was calculated approximately 75 degrees from the coronal plane (15 degrees off-sagittal) with a magnitude of between 10 and 11 degrees.

A focal dome was chosen to correct the varus recurvatum and translation. The surgical approach was an anterior approach over the anterior compartment, elevating the tibialis anterior from the lateral tibia to allow safe access to the plane of the deformity. The fibula was osteotomized near the level of the tibia through a separate lateral approach. The plane of maximum deformity was assessed intraoperatively and confirmed the preoperative calculations. The ‘no deformity’ view on fluoroscopy was located, with the maximum plane orthogonal to this. It is in this plane that the drill bit at the CORA is directed, and this is especially important if correcting a translation. The arc of the osteotomy was drawn using a parallel drill guide with one bit centered at the CORA (Figure 7C).

The angulation and translation were simultaneously corrected. Dual medial–lateral plates were used to counteract the lateral gap forming with medial plate compression. Hence, balanced compression from the medial and lateral plates restored the alignment and provided absolute stability (Figure 7D). The patient had immediate relief of knee pain after correction. The soft tissue and osteotomy healed without incident (Figure 7E).

### 3.3. Case 3-Single-Plane Correction with Nail: Tibia

**Figure 8:**
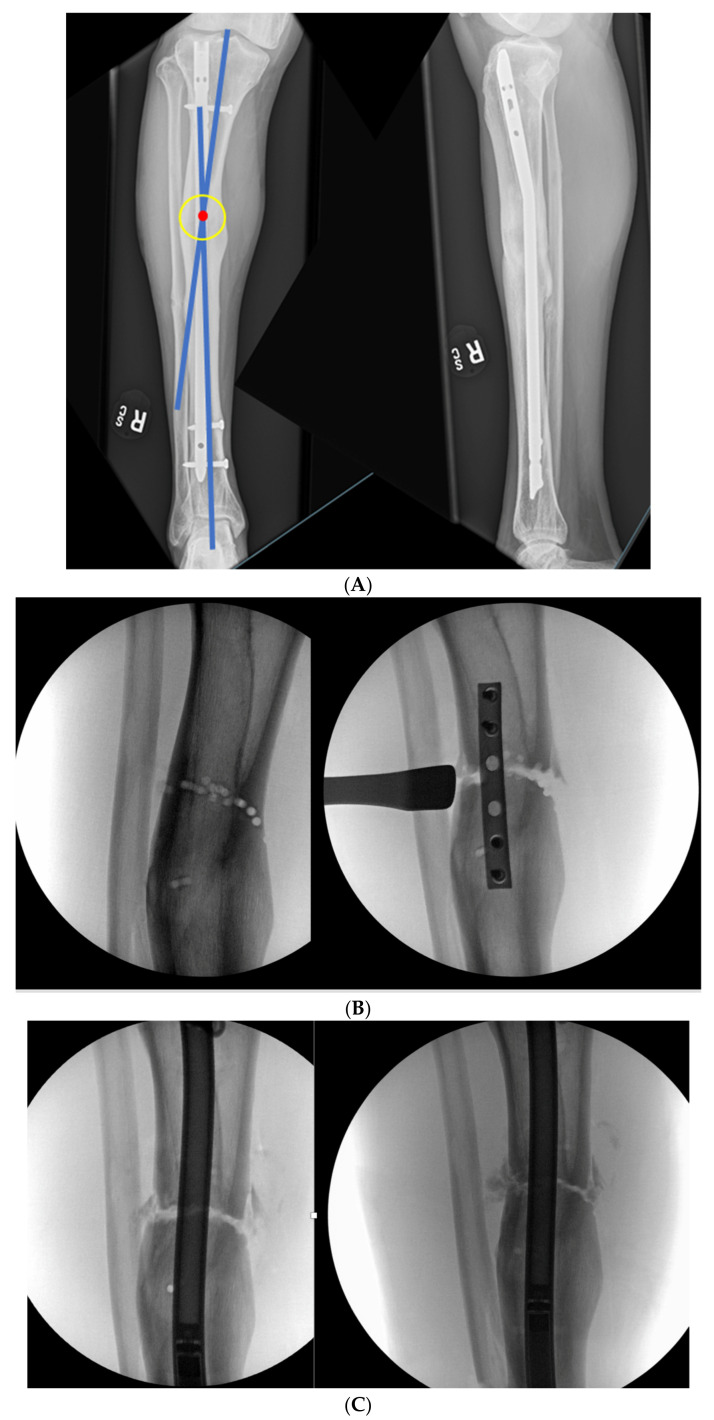

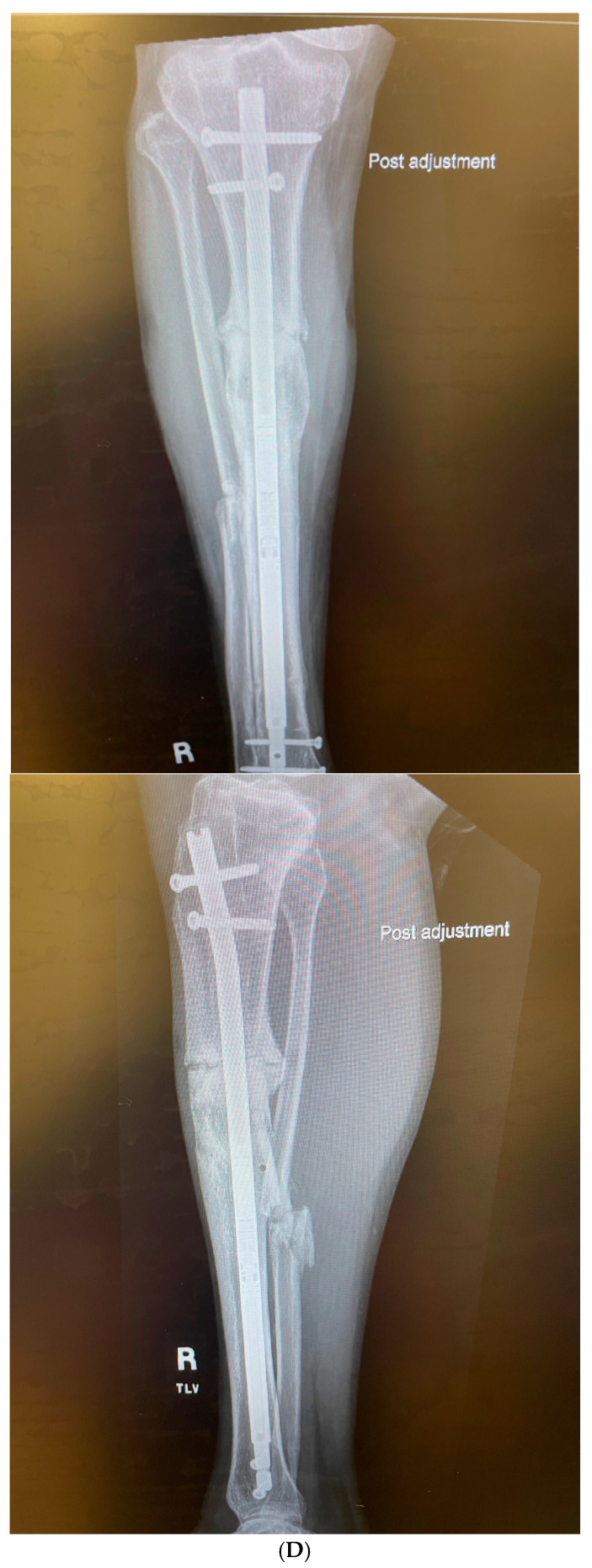
(**A**) Adult male with varus malunion deformity after a prior tibia fracture and nailing. (**B**,**C**) Patient underwent focal dome osteotomy with compression nailing using a magnetic motorized nail for early and subsequent nail compression. (**D**) Patient declined to follow-up after 12 weeks, citing absence of pain. Patient returned at 6 months at request of surgeon, still without pain. Magnetic nail recompressed. Patient lost to follow-up after 6 months.

An adult male with no comorbidities presented with varus malunion following intramedullary tibial nailing, reporting symptomatic medial knee pain (Figure 8A). The CORA analysis revealed a varus deformity of 12 degrees. With a deformity in the coronal plane, surgical access was through an anterior approach using a focal dome osteotomy for correction. The anterior compartment was elevated, and a drill bit was placed in the CORA, with a parallel drill guide used to draw the arc with a second drill bit. The osteotomy was completed with an osteotome. The alignment was maintained during reaming using a temporary plate (Figure 8B). The patient underwent compression nailing using a magnetic motorized nail, allowing for subsequent compression as needed after discharge (Figure 8C). The patient felt pain-free at 12 weeks, yet was encouraged to return for regular follow-up examinations and X-rays until radiographic healing. Patient lost to follow-up after 6 months (Figure 8D).

### 3.4. Case 4-Infected Malunion-Nonunion Femur Correction: True Dome Osteotomy

**Figure 9:**
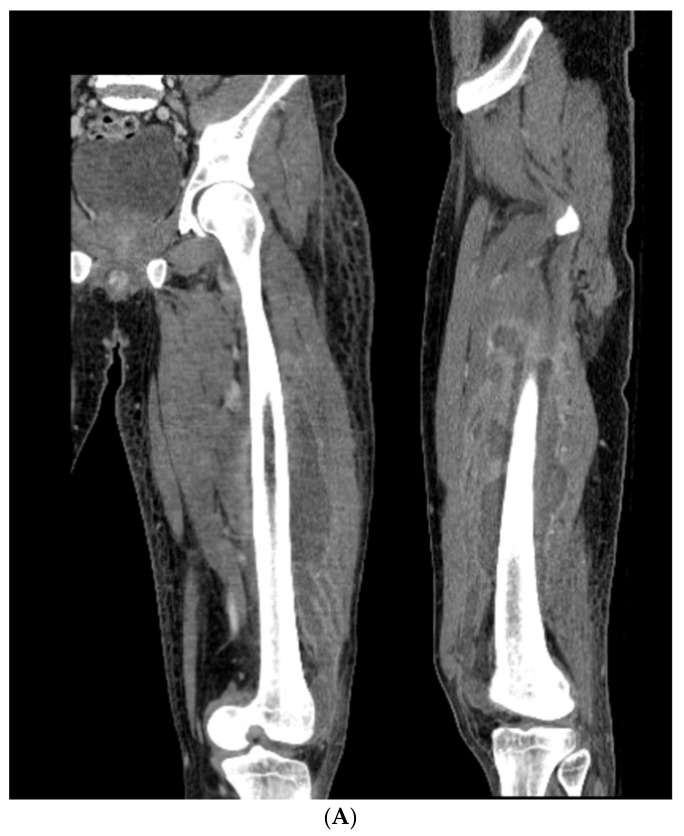

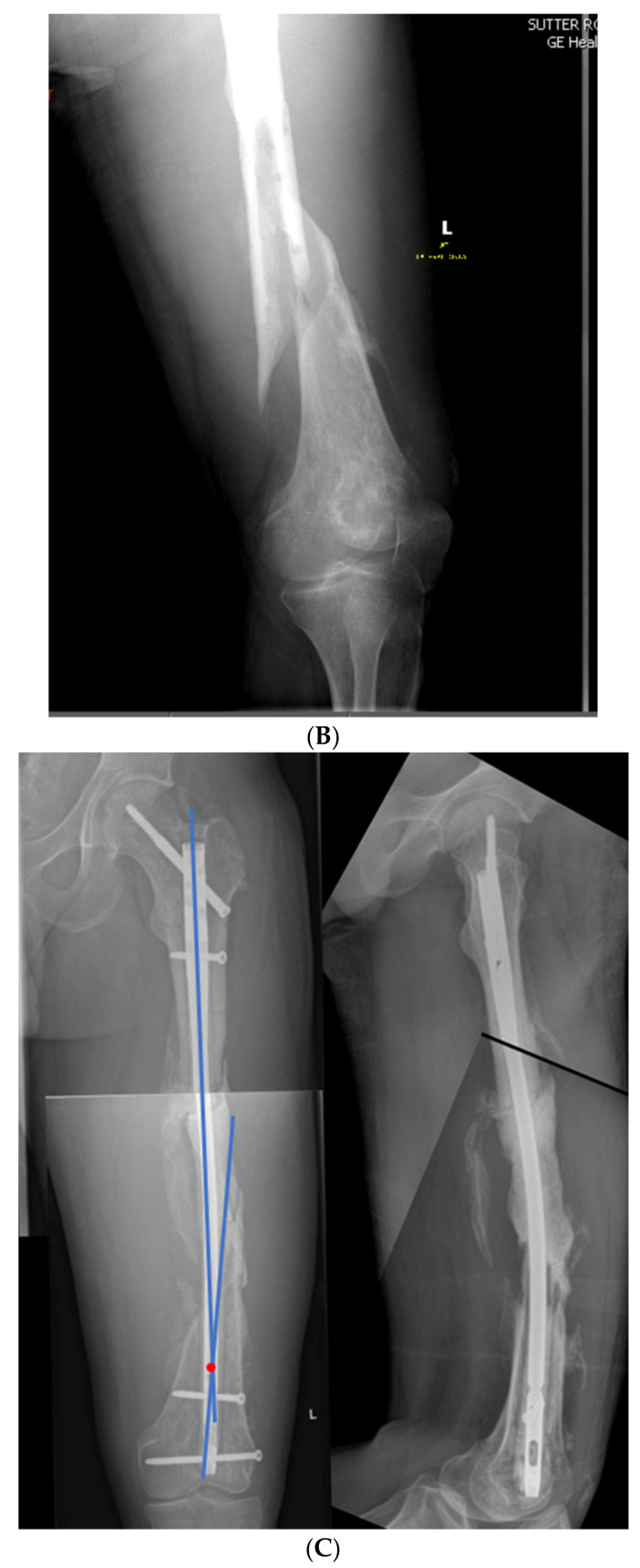

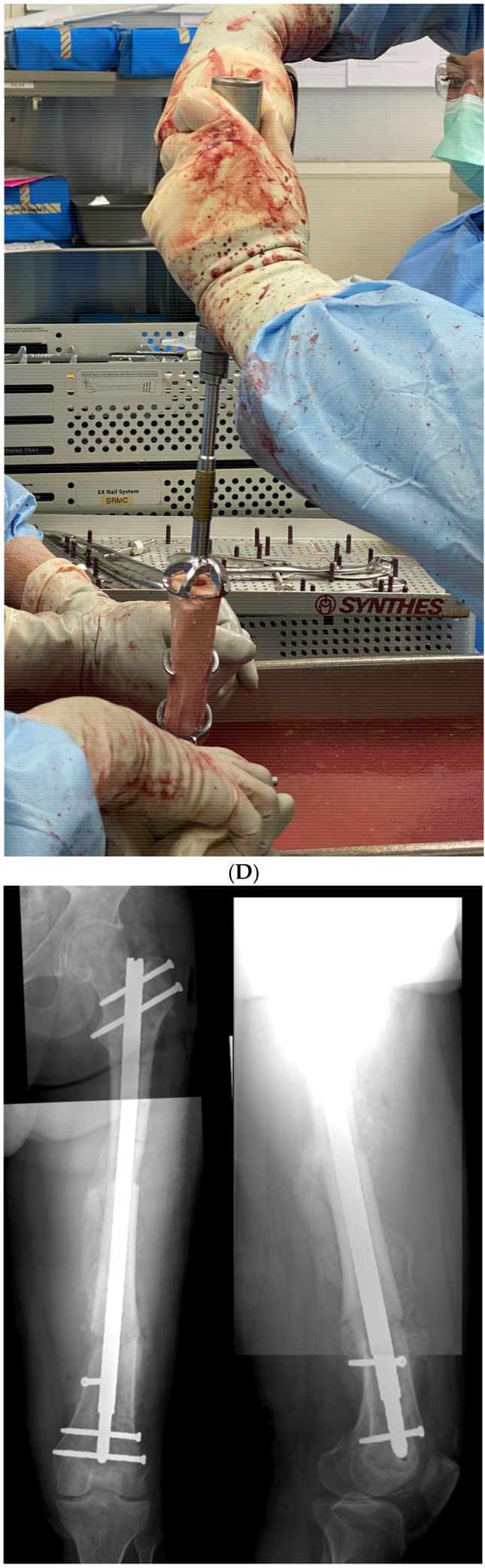

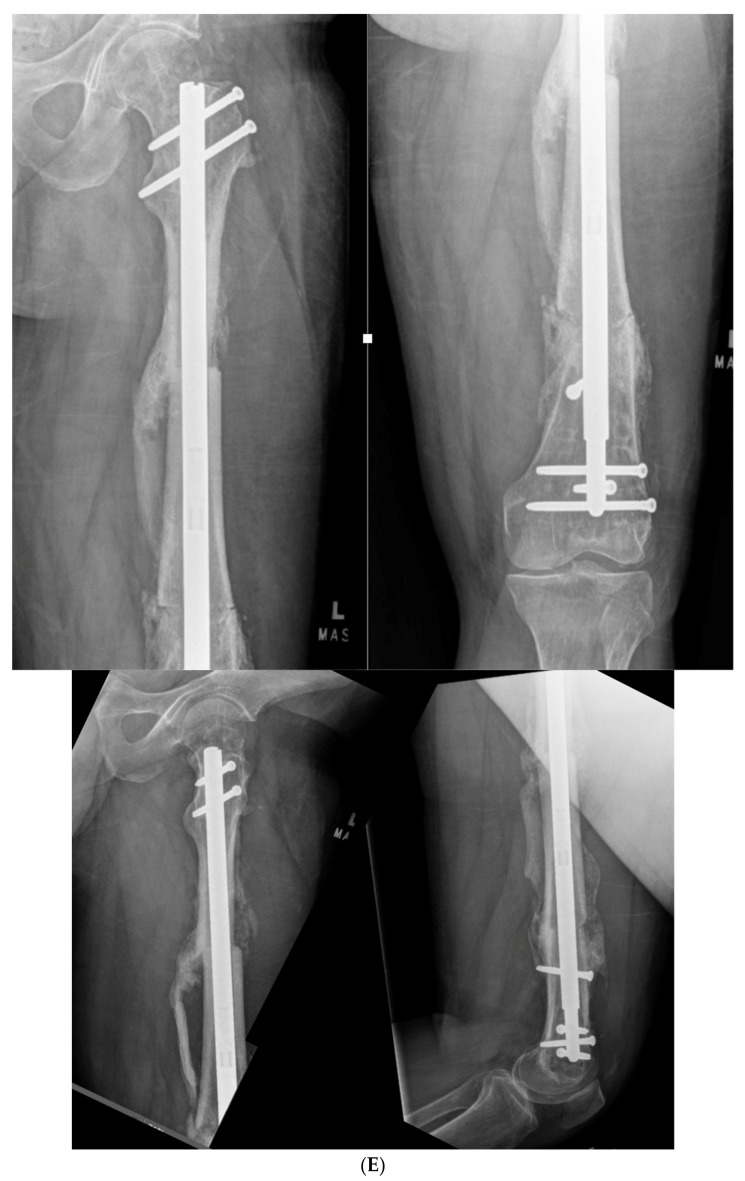

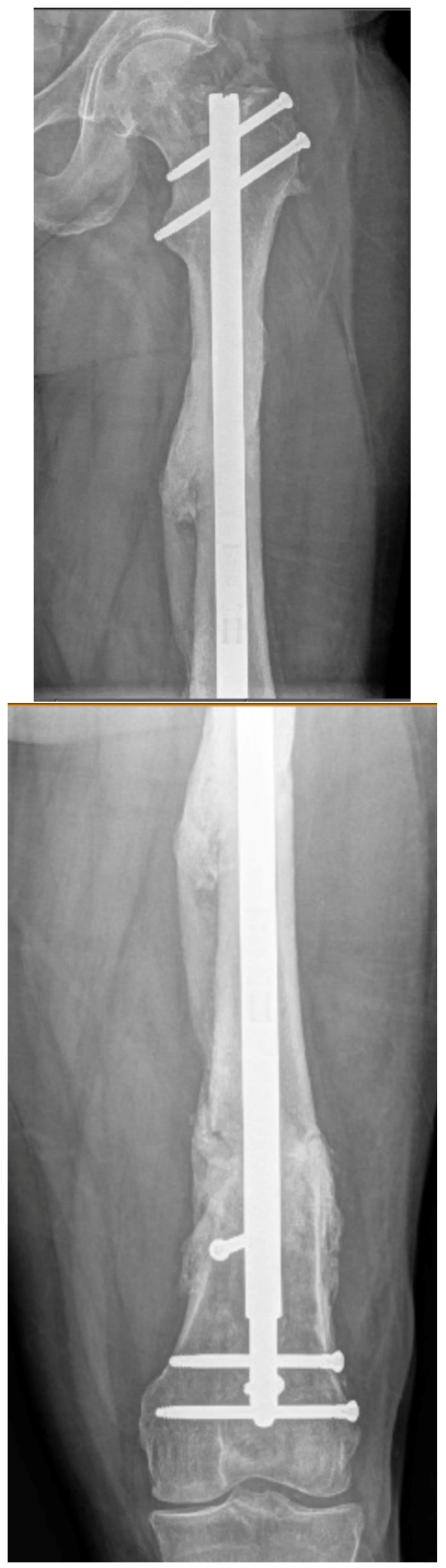

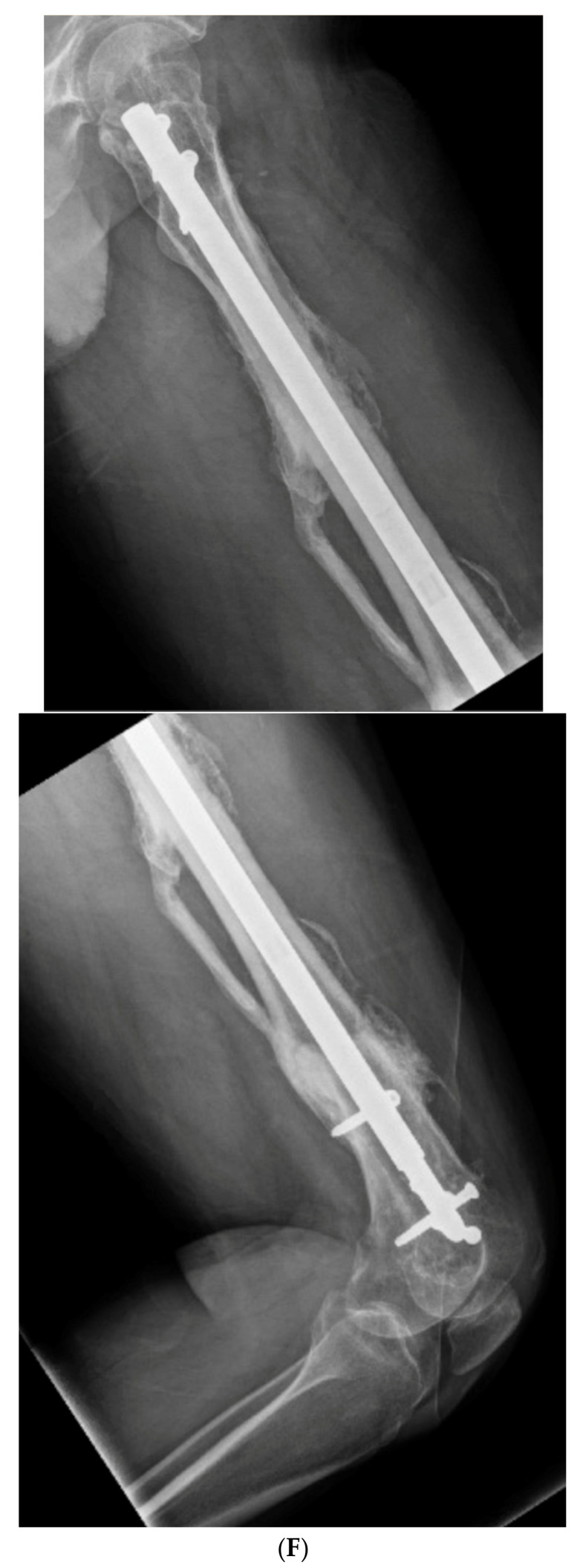
(**A**) A young adult male presented new-onset diabetic ketoacidosis with sepsis. He was found to have a large spontaneous left thigh abscess, as seen on CT imaging. (**B**) Multiple surgeries resulted in a femur fracture and subsequent segmental resection of approximately 13 cm of the patient’s femoral diaphysis. (**C**) The patient eventually attained source control with resection, antibiotic spacer, and an antibiotic coated nail. Patient has a 5-degree distal varus deformity. (**D**) The patient underwent a true dome osteotomy of the proximal and distal ends of the allograft/host bone interfaces. Fresh frozen allograft preparation using a reverse reamer is shown prior to implantation. (**E**) Motorized compression of the construct was employed with early and subsequent compression. Four-month follow-up showed restored neutral alignment, incorporation/interface healing of the dome osteotomy sites, bone regenerate across the interfaces, normal lab markers, and return to gainful employment. (**F**) Twelve-month follow-up continued to demonstrate interface healing without infection recurrence.

A young adult male presented new-onset diabetic ketoacidosis and sepsis. The study revealed a large thigh abscess circumferentially surrounding the left femur (Figure 9A). No other source was identified during workup. The patient underwent multiple debridements, and initially, a cortical window was made for the egress of intramedullary purulence. Subsequent debridements revealed extensive osteomyelitis. During debridement with intramedullary antibiotic nailing, the femur fractured and was eventually resected to attain source control (Figure 9B). A 13-cm segmental defect was stabilized using an antibiotic-coated locked nail with the surrounding antibiotic bone cement spacer Figure 9C). The CORA analysis revealed a five-degree varus deformity and no significant sagittal plane deformity. After source control with intravenous antibiotics and a 3-month course of oral antibiotics, the workup, including a biopsy and culture, resulted in no growth and no sign of ongoing infection. A bifocal true dome osteotomy with intercalary cortical allograft was chosen based upon a low likelihood of compliance with bone transport. A standard lateral approach elevating the vastus lateralus was used to access the femur. Reverse reamers and standard acetabular reamers were used for mating the bone surfaces (Figure 9D). The varus alignment was addressed using a blocking screw as the nail was passed; no gap was produced at the osteotomy due to its true dome shape. The dome osteotomy interfaces were autografted and compressed with a magnetic motorized nail, with additional compression at the subsequent outpatient visits (Figure 9E). The patient had soft tissue and allograft interface healing without incident and returned to gainful employment (Figure 9F).

## 4. Complications

Complications specific to focal dome osteotomy of the diaphysis include nonunion requiring bone grafting. The deformity analysis, while not complex in concept, is required for properly establishing the central point for the radius of the osteotomy. The diaphyseal techniques discussed herein with the case reports illustrated the ability to safely and fully correct the deformities while also demonstrating the potential need for bone grafting. No wound or soft tissue complications were encountered due to acute correction. All patients achieved and maintained correction without recurrence of a pre-existing infection. 

## 5. Discussion

Most literature regarding focal dome osteotomies arises from metaphyseal corrections, where the results are comparable to wedge osteotomies [12]. In fact, opening wedge osteotomies are quite well-tolerated in the metaphysis [2,5]. The simplest form of osteotomy should be used when possible for correction. In the metaphysis for most extraarticular cases, a wedge osteotomy is preferred for this reason. The simplicity of a focal dome, however, is in the ability to adjust angulation intraoperatively. The dome osteotomy also allows for large amounts of compression and inherent stability, which, for many cases, is preferable, especially in the diaphysis. The opening wedge is not recommended due to concern for fixed gaps that impair bone healing in the diaphysis. Closing wedge osteotomy is a very viable option in the diaphysis [13]. Wedge osteotomy also requires meticulous deformity analysis and execution and is compressible. However, an additional layer of accuracy is required to remove exactly the degree of wedge intended to produce an accurate deformity correction. The removal of bone with a closing wedge will shorten the limb, whereas focal dome osteotomy will not. Another option for complex diaphyseal deformity is the clamshell osteotomy [14]. The clamshell is superior for long curved defects and those that also require rotational correction. The clamshell also avoids determination of the CORA and its exact placement for the center of a focal dome correction. The focal dome cannot address axial rotation but can accommodate translation; the true dome osteotomy is capable of rotational corrections with its more extensive exposure [9]. The dome osteotomies in the diaphysis require more meticulous attention to detail when executing the osteotomy with sharp drill bits, low-energy cuts, and copious irrigation. Failure to apply this level of care may result in nonunion due to thermal injury. With intramedullary fixation, the nail path must exactly match the axis of the proximal and distal diaphysis (this applies to a closing wedge as well) and requires more attention to detail with reaming than an opening wedge osteotomy or an extramedullary fixation technique such as plating. Ultimately, the surgeon must be facile with the CORA analysis to succeed with most of the osteotomies discussed. The focal dome is a satisfying compromise; it requires meticulous planning like a closing wedge, but once the cut is made, it is flexible in attaining the exact correction desired. Compressing and maintaining stability is outstanding for diaphyseal deformity unless the execution of the osteotomy is imperfect.

### Future Directions

Applying the deformity correction using improved arcuate cutting devices will facilitate a more accurate and compressible focal dome osteotomy. The true dome currently necessitates the use of standard dome-shaped reamers. Improved matching concave and convex reamers will facilitate true dome osteotomy with a smaller biological footprint.

## 6. Conclusions

In the author’s experience, focal and true dome osteotomy are viable options to include in the array of techniques used for diaphyseal malunions of the lower extremities. As with any surgical correction in the diaphysis, bone healing requires absolute attention to detail to maximize the biological capacity of the cut ends. The deformity analysis is relatively simple and uses the CORA method, widely understood by many surgeons who perform corrective osteotomies. Surgeons who have experience with diaphyseal wedge osteotomy and clamshell osteotomy will find the focal dome osteotomy to be of a similar difficulty level, yet it provides another option for fine-tuning the alignment intraoperatively.

## Data Availability

The data and patients presented in this study are available upon request from the corresponding author. The data is not publicly available due to patient confidentiality.

## Supplementary Materials

The following are available online at https://www.mdpi.com/article/10.3390/medicina58020308/s1: Video S1: demonstration of dual drill bit technique for drawing focal dome osteotomy. Video S2: Use of reverse reamer for creating mating true dome osteotomy surfaces.

## References

[1] Dabis J., Templeton-Ward O., Lacey A.E., Narayan B., Trompeter A. (2017). The history, evolution and basic science of osteotomy techniques. Strateg. Trauma Limb Reconstr..

[2] Paley D. (2002). Principles of Deformity Correction.

[3] Tunggal J.A.W., Higgins G.A., Waddell J.P. (2010). Complications of closing wedge high tibial osteotomy. Int. Orthop..

[4] El Ghazaly S.A., El-Moatasem E.-H.M. (2015). Femoral supracondylar focal dome osteotomy with plate fixation for acute correction of frontal plane knee deformity. Strateg. Trauma Limb Reconstr..

[5] Marti R.K., van Heerwaarden R.J. (2008). Osteotomies for Posttraumatic Deformities.

[6] Russell G.V., Graves M.L., Archdeacon M.T., Barei D.P., Brien G.A., Porter S.E. (2009). The clamshell osteotomy: A new technique to correct complex diaphyseal malunions. J. Bone Jt. Surg..

[7] Gaston M.S., Simpson A.H.R.W. (2007). Inhibition of fracture healing. J. Bone Jt. Surg. Br. Vol..

[8] Rozbruch S.R., Fragomen A.T., Ilizarov S. (2006). Correction of tibial deformity with use of the Ilizarov-Taylor spatial frame. J. Bone Jt. Surg..

[9] Wilke B., Cooper A., Gibbs C.P., Spiguel A. (2018). Reverse-reamed intercalary allograft: A surgical technique. JAAOS-J. Am. Acad. Orthop. Surg..

[10] Patterson F.R., Hwang J.S., Beebe K.S., Uglialoro A.D., Flynn J., Benevenia J. (2012). An innovative approach to concave–convex allograft junctions: A biomechanical study. Am. J. Orthop..

[11] Bastien N., Kelly S., Lybeck D. (2021). Concave-Convex Reaming of Intercalary Allograft: 1-year Clinical Outcomes. JAAOS Glob. Res. Rev..

[12] El-Rosasy M., Ayoub M. (2007). Acute correction of proximal tibial deformities in adolescents using Ilizarov external fixator: Focal-dome versus straight-cut osteotomy. J. Pediatric Orthop. B.

[13] Santoro D., Tantavisut S., Aloj D., Karam M.D. (2014). Diaphyseal osteotomy after post-traumatic malalignment. Curr. Rev. Musculoskelet. Med..

[14] Purcell K.F., Russell G.V., Graves M.L. (2021). The Clamshell Osteotomy for Diaphyseal Malunion in Deformity Correction and Fracture Surgery. Medicina.

